# Designing Patient-Centered Interventions for Emergency Care: Participatory Design Study

**DOI:** 10.2196/63610

**Published:** 2025-02-12

**Authors:** Woosuk Seo, Shruti Jain, Vivian Le, Jiaqi Li, Zhan Zhang, Hardeep Singh, Kalyan Pasupathy, Prashant Mahajan, Sun Young Park

**Affiliations:** 1 School of Information University of Michigan Ann Arbor, MI United States; 2 Computer Science and Engineering University of Michigan Ann Arbor United States; 3 Seidenberg School of Computer Science and Information Systems, Pace University New York, NY United States; 4 Center for Innovations in Quality, Effectiveness and Safety (IQuESt), Michael E. DeBakey Veterans Affairs Medical Center and Baylor College of Medicine Houston, TX United States; 5 Biomedical and Health Information Sciences, University of Illinois Chicago Chicago, IL United States; 6 Department of Emergency Medicine, University of Michigan Medical School Ann Arbor, MI United States; 7 School of Information, Stamps School of Art and Design, University of Michigan Ann Arbor, MI United States

**Keywords:** emergency department, participatory design, patient, technology, intervention

## Abstract

**Background:**

Emergency departments (EDs) are high-pressure environments where clinicians diagnose patients under significant constraints, including limited medical histories, severe time pressures, and frequent interruptions. Current ED care practices often inadequately support meaningful patient participation. Most interventions prioritize clinical workflow and health care provider communication, inadvertently overlooking patients’ needs. Additionally, patient-facing technologies in EDs are typically developed without meaningful patient input, leading to solutions that may not effectively address patients’ specific challenges. To enhance both patient-centered care practices and the diagnosis process in EDs, patient involvement in technology design is essential to ensure their needs during emergency care are understood and addressed.

**Objective:**

This study aimed to invite ED patients to participatory design sessions, identify their needs during ED visits, and present potential design guidelines for technological interventions to address these needs.

**Methods:**

We conducted 8 design sessions with 36 ED patients and caregivers to validate their needs and identify considerations for designing patient-centered interventions to improve diagnostic safety. We used 10 technological intervention ideas as probes for a needs evaluation of the study participants. Participants discussed the use cases of each intervention idea to assess their needs during the ED care process. We facilitated co-design activities with the participants to improve the technological intervention designs. We audio- and video-recorded the design sessions. We then analyzed session transcripts, field notes, and design sketches.

**Results:**

On the basis of ED patients’ feedback and evaluation of our intervention designs, we found the 3 most preferred intervention ideas that addressed the common challenges ED patients experience. We also identified 4 themes of ED patients’ needs: a feeling of inclusion in the ED care process, access to sources of medical information to enhance patient comprehension, addressing patient anxiety related to information overload and privacy concerns, and ensuring continuity in care and information. We interpreted these as insights for designing technological interventions for ED patients. Therefore, on the basis of the findings, we present five considerations for designing better patient-centered interventions in the ED care process: technology-based interventions should (1) address patients’ dynamic needs to promote continuity in care; (2) consider the amount and timing of information that patients receive; (3) empower patients to be more active for better patient safety and care quality; (4) optimize human resources, depending on patients’ needs; and (5) be designed with the consideration of patients’ perspectives on implementation.

**Conclusions:**

This study provides unique insights for designing technological interventions to support ED diagnostic processes. By inviting ED patients into the design process, we present unique insights into the diagnostic process and design considerations for designing novel technological interventions to enhance patient safety.

**International Registered Report Identifier (IRRID):**

RR2-10.2196/55357

## Introduction

### Background

In the high-pressure environment of emergency departments (EDs), diagnosing and managing patients is inherently complex. This complexity arises from extensive interactions among various stakeholders, including health care providers, family caregivers, and patients. The dynamic and time-sensitive context of EDs forces stakeholders to make critical medical decisions promptly. The urgent nature of emergency care thus poses substantial challenges to ED providers, compounding the difficulty of achieving timely and accurate diagnoses and raising concerns regarding patient safety. While exact rates of diagnostic errors remain elusive, a conservative estimate of 5% errors in adults out of the 131 million annual ED visits translates to about 7 million cases of ED-based diagnostic errors [1]. Notably, nearly half of these diagnostic errors have the potential to cause harm to patients.

Many existing technological interventions focus on supporting the diagnostic process and promoting patient safety in the ED. For example, an ED provider-facing mobile app currently exists to deliver real-time patient information and allows for collaborative patient management for care teams [2]. Artificial intelligence (AI)–based software [3] has also been developed to provide practical guidance to novice nurses or scan operators in the ED who have limited experience in echocardiography. In addition, AI-based systems [4,5] have been used to support documentation for the nursing team, indirectly promoting the efficiency of the care process. Such systems could support providers by easing the documentation burden (eg, parsing long notes and automatically populating relevant text) so that they could focus more on patient care.

On the other hand, some technological interventions have been developed that focus more on ED patients. An indoor navigation system [6] was developed to assist patients unfamiliar with the ED environment, and a mobile display [7] was designed to present an interactive report detailing patients’ progress, care plans, and care teams throughout their stay in the ED. To reduce patients’ anxiety, some prior studies highlighted opportunities to use music apps [8] or virtual reality applications [9]. More recently, a study presented the opportunity for an AI-based application to provide medical triage to patients more efficiently [10]. Compared with human clinicians, the AI-based application made probable triage decisions. While these technological interventions improve providers’ diagnosing work or patient safety, direct input from patients and providers working at the frontline is often overlooked when developing such solutions.

Participatory design (PD) [11] is a methodology that engages all stakeholders in the design process to create solutions that address their needs. PD has been widely used by researchers in prior studies to co-design user-centered technologies with health care providers. For instance, Kusunoki et al [12] conducted PD workshops with trauma team members to understand the different needs of awareness support among the various roles of team members and identify concrete design strategies to manage these differences. Pollack et al [13] organized a design session with 11 clinicians to develop a clinical information tool using PD techniques. On the basis of the session’s findings, the authors identified benefits (eg, a high level of domain knowledge can be used to anticipate how design ideas can be applied to clinical processes and workflow) and potential challenges (eg, power dynamics between physicians) of leveraging PD techniques in designing a clinical information tool. The authors also outlined guiding principles for implementing these methods in health care organizations interested in advancing health information technology. These prior studies have presented how PD is helpful and practical for designing human-centered technology in health care settings.

Despite the benefits of PD in designing health technology, limited work has adopted a user-centered PD approach to develop technological interventions for ED specifically to enhance patient safety. Østervang et al [14] conducted PD workshops with health care providers and patients to design an ED information system. In the study, providers brainstormed initial design ideas for an ED information system in a workshop; then, patients provided feedback in separate workshops. The authors presented how the PD approach helps yield insights from ED providers and patients to create a more user-centered system. However, ED patients in the study had limited participation because the workshops were conducted one-on-one, and patients were only asked to provide feedback on intervention ideas developed by health care providers.

### Objectives

Building on previous research, we aimed to engage ED patients in validating their needs and co-designing novel interventions to promote patient safety and enhance the ED diagnostic process. Because patients are vulnerable to potential errors, their involvement in developing patient-centered interventions is important to improve the diagnostic process. Through identifying patients’ needs during their ED visits, this study presents design guidelines for more patient-centered technological interventions. We used preliminary technological intervention concepts to facilitate discussions about participants’ experiences and needs. This approach helped expand our understanding of patient experiences in the ED. Rather than proposing specific solutions, we aimed to refine intervention concepts based on patient input and establish broader guidelines for designing patient-facing interventions.

## Methods

### Overview

This study is part of a larger project that aims to identify ED patients’ and health care providers’ perspectives on technological interventions to support the ED diagnostic process and to craft design guidelines for interventions that meet both stakeholders’ needs. In this paper, we focus on validating the needs of ED patients and caregivers during ED visits and their perspectives on technological interventions. We conducted 8 design sessions with 36 ED patients or their caregivers. On the basis of the analysis of transcripts, design sketches, and field notes, we identified 4 themes representing patient needs, strategies to mitigate common challenges, and design guidelines for potential interventions.

### Design Idea Generation Phase for ED Care Interventions

**Figure 1:**
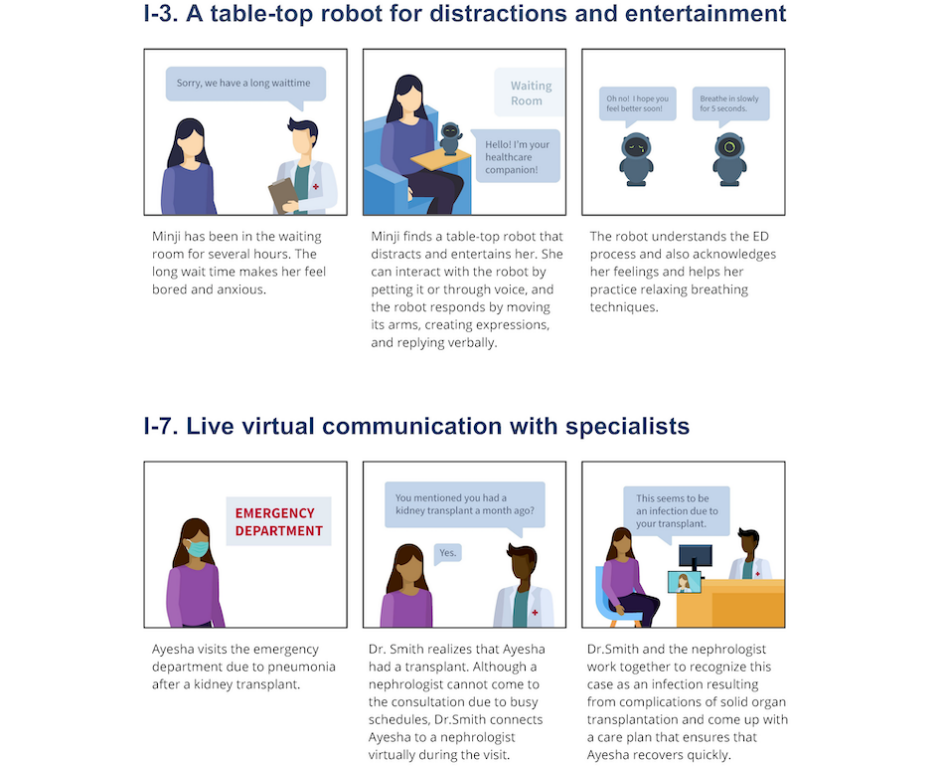
Two sample storyboards of technological intervention ideas presented to the participants during the sessions (top: table-top robot; bottom: virtual specialist). ED: emergency department.

Before our PD study with ED patients, the research team interviewed 8 patients and caregivers to better understand their experiences and challenges during ED visits. From the interviews, we identified difficulties, emerging patterns of complaints, and general levels of satisfaction with different aspects of the care process (Table 1). Common themes were discovered from each problem category through analysis of the interview data ranging from patient and caregiver difficulties, complaints, and satisfaction levels with various aspects of the ED care process. In addition to the known problems, such as long wait times and insufficient or poor communication with providers, ED patients and caregivers also faced difficulties because of the absence of caregivers, lack of ED process literacy, or inadequate understanding of discharge information. On the basis of the findings, the research team brainstormed numerous design ideas for each problem category, focusing on the stakeholders (eg, patients, caregivers, and providers). We then merged the design ideas based on feasibility and usefulness. Finally, we narrowed the list and finalized the 10 most effective intervention ideas (Table 2). These ideas were used to validate patient needs during each PD session, presented as storyboards, as shown in Figure 1. Each intervention idea aims to address at least one problem category.

**Table 1:** A list of identified problem categories from the previous study’s patient interview data.

Problem category	Examples
Waiting room general challenges	Patients feel forgotten in the waiting room because health care providers do not check up on them Patients are left in pain in the waiting room
Overcrowding challenges	There are patients with mild symptoms who make the ED^a^ more crowded
Information presentation and overload challenges	Patients are inadequately informed of the ED process during the visit (ie, lack of ED process literacy) Test results can be confusing for patients to understand because of the use of medical terminology
Information sharing challenges	Patients often feel that there are errors in the physician’s notes (eg, it is different from what was discussed in the visit) Health care providers at hospitals sometimes wait for information transfer from the patient’s original hospital, which can take time
Communication challenges	Patients find it hard to recount all personal medical details in the ED because they are sick, stressed, or unaware Patients sometimes have trouble communicating with providers because of challenges like being a nonnative English speaker
Contagious disease transfer in ED	Crowded waiting rooms can have many sick patients in close proximity, which makes patients concerned Contact with surfaces can cause disease transfer
Discharge and postvisit challenges	Lack of postvisit resources for patients Patients feel that they have been wrongly discharged

^a^ED: emergency department.

As probes, the storyboards incorporated user requirements and insights about patient-centered interventions to help participants better understand the use cases and contexts where the proposed interventions could be implemented. Each storyboard consisted of a 3-panel illustration, describing an example of a patient facing one of the challenges mentioned above and using the intervention to mitigate or prevent the problem (see Figure 1 for sample storyboards). Details of the intervention’s features and functionality were left vague. We wanted participants to focus on the need rather than specific features to encourage more discussion about improving the intervention concept. Each storyboard included a lead question to highlight the user’s needs (ie, patients) outlined in each intervention and to facilitate participant discussion about whether they had experienced the need, rather than directly referencing the intervention. Using storyboards allowed participants to have less biased and more open-ended discussions in the PD sessions. Table 2 describes each technological intervention and the patient need it addresses, along with storyboard samples.

**Table 2:** A list of 10 intervention ideas with brief descriptions and discussion questions to validate patient needs.

Intervention number	Technological intervention	Description	Sample of lead question used for the discussion of patient need
I-1	Virtual presence of a caregiver	A communication device for ED^a^ patients that enables the virtual presence of the caregiver for the patient and their ED provider when the caregiver cannot physically be with the patient	Have you experienced any difficulty visiting the ED alone without a caregiver?
I-2	Transparent interactive display for ED-related guidance	An interactive display for ED patients in a waiting room that displays ED-related guidance and acts as a separator between patients for personal hygiene	During your ED visit, have you worried that you might be coming in contact with contagious diseases while in the waiting room?
I-3	Table-top robot for distraction and entertainment	A table-top robot for ED patients in the waiting room that provides distraction and entertainment as they wait	Have you felt distraction and entertainment is helpful during a long wait in the ED?
I-4	Wearable device for monitoring real-time condition	A wearable device for ED patients in the waiting room that tracks their real-time data (eg, vitals, such as heart rate and blood oxygen) and notifies providers if vitals indicate a patient needs immediate help	Have you felt worried about your condition worsening while waiting, and being unable to inform healthcare providers properly?
I-5	Assistant robot in the waiting room	A mobile robot that assists and checks on ED patients in the waiting room	Have you felt forgotten while waiting because of busy ED providers not checking up on your condition?
I-6	ED-specific chatbot for ED practice literacy	A mobile device–compatible chatbot that can provide ED-specific information directly from the hospital to patients	Have you ever felt confused about the meaning of the medical information you received during ED care?
I-7	Virtual communication with a specialist	A communication device for ED patients and providers that enables a virtual consultation with a specialist during ED care	Have you felt the need to consult with a specialist during your ED visit?
I-8	Pain expression device	A tactile device that helps ED patients understand and describe pain through vibrations and haptic feedback	Have you had any difficulties in describing your pain verbally?
I-9	Patient-facing screen for sharing physician’s notes	A screen display that presents care-related information for patients to view during consultation or follow-up with providers about diagnosis or test results	Have you ever noticed errors or confusing details on physician’s notes (usually on discharge notes or after-visit summaries)?
I-10	AI^b^ caller for providing after-visit support	A follow-up call powered by AI to help answer patients’ postdischarge questions regarding their ED visit	Have you had any challenges or questions that needed to be properly addressed after your ED visit?

^a^ED: emergency department.

^b^AI: artificial intelligence.

### Participant Recruitment

We recruited patients and caregivers who had visited the ED within 6 weeks from the point of contact. Participants were mainly recruited from the adult and pediatric EDs at a university-affiliated hospital in 3 ways: calling or emailing them after their most recent ED visit, posting fliers with the study’s contact information in the adult ED and the pediatric ED, and approaching patients during their ED visit. The inclusion criteria for participants were as follows: (1) aged ≥18 years old, (2) previously visited ED within 6 weeks from the point of contact, and (3) comfortable speaking English. During a patient’s ED visit, researchers mainly focused on recruiting those having a triage level of 3 to 5, with 1 being critical and 5 being nonurgent, to avoid disrupting or risking their care. We contacted more than 150 potential participants via email and phone, as participant retention was challenging because of last-minute cancellations from patients. In total, 36 ED patients and caregivers participated in 8 design sessions (see Table 3 for their demographic information). All but one session had 4 or 5 participants, and one session had 3 participants. As compensation for participation, each participant was provided with a US $100 gift card for the session.

**Table 3:** Demographic information of study participants (N=36).

Participant demographics		Participants, n (%)
**Race**		
	Asian	1 (3)
	Black	7 (19)
	White	27 (75)
	Unknown	1 (3)
**Sex**		
	Male	12 (33)
	Female	24 (67)
**Participant type**		
	Patient	23 (64)
	Caregiver	13 (36)

### PD Session Procedure

We conducted 8 PD sessions. Each session was roughly divided into three parts: (1) session introduction, (2) storyboard critique, and (3) co-design as a group. Each session took approximately 2 hours.

**Figure 2:**
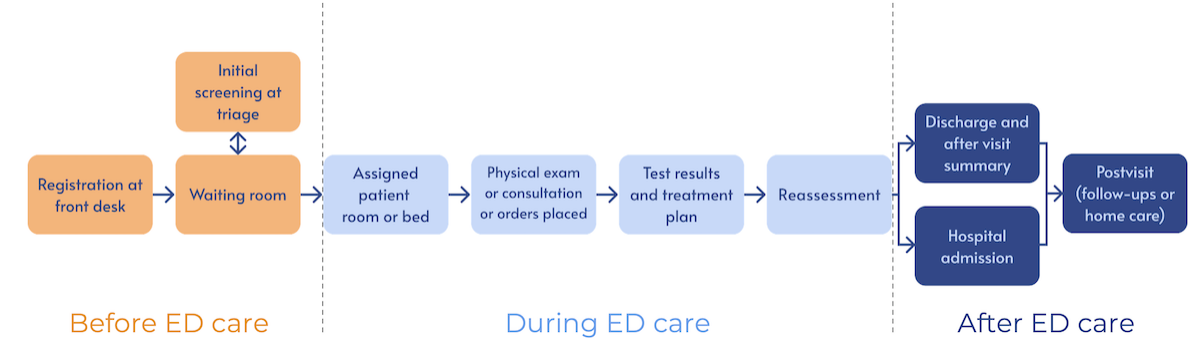
A timeline of the emergency department (ED) care process illustrating steps before, during, and after care. This timeline, based on prior studies, was provided to the participants as a reference for recalling their ED visits.

In the introductory session, participants were each given a paper packet outlining the current ED care process timeline, the 10 technological intervention storyboards for reference, and a sheet to rank the interventions based on their preferences. A presentation of the ED care process timeline (Figure 2) was shown to prompt participants to recall their previous or most recent ED experience. We created this timeline based on our last interview study [15] and prior studies on the ED care framework [16]. We first asked participants to write down one major challenge they encountered during this experience and specifically indicate where it occurred in the current ED care process timeline in their packet. Each participant was then asked to discuss their experiences with the group, establishing the foundation for the main session activities. Allowing participants to reflect on their previous or most recent ED visit prefaced discussions about how their challenges might or might not be addressed with one or more of the 10 technological interventions and the co-design activity.

For the storyboard critique activity, 10 intervention design ideas were presented via storyboards to prompt participant discussion about each intervention’s feasibility in the current ED care process. Researchers encouraged participants to initiate the conversation with lead questions and additional follow-up questions related to each scenario were asked, as necessary, to maintain the discussion within the group. The discussion of the 10 technological interventions provided a space for participants to speak about their experiences and needs during ED visits and for researchers to better understand the underlying challenges patients face. After all the intervention storyboards were presented, participants ranked their top 3 preferred interventions based on preference and potential feasibility in ED care. While the main task for each participant was to consider the interventions from the patient or caregiver perspective, participants naturally framed these interventions from the provider’s perspective as well. We did not set any restrictions regarding this task during the discussions and design activities to solicit as much feedback as possible regarding the feasibility of each intervention in the ED care context.

**Figure 3:**
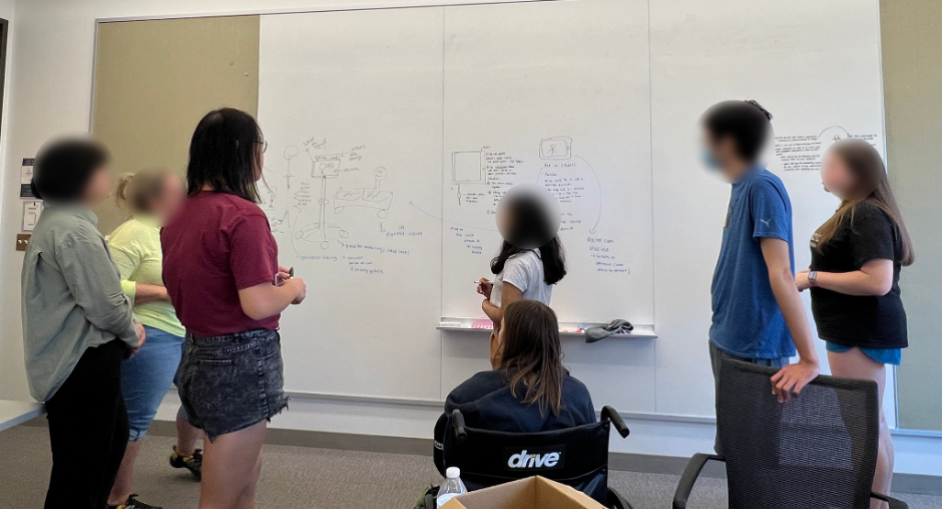
Co-design activity with the participants in the middle of a discussion about improving idea 7 (I-7 in Table 2). Participants collaboratively drew ideas on a whiteboard and wrote notes about imagined functionality.

Each group of participants discussed their most preferred 3 interventions and then selected one for the co-design activity. Then, as a group, participants had approximately 25 to 30 minutes to augment their chosen technological intervention to improve the ED experience (Figure 3). Participants were tasked with adding, changing, and removing features or modifying the context of the technological intervention to help it better align with their preferred, ideal future implementation. Researchers emphasized the importance of focusing on the roles and functionality of technological interventions rather than the aesthetic quality of the designs. The co-design activity helped us understand the features of the intervention technologies participants desired to address patient needs, along with other fundamental and feature-specific aspects that may not have been revealed in the storyboard critique. Through this, participants could also discuss their decisions in detail, out loud, and reach a consensus about the most critical design features to incorporate into an ideal intervention solution. Following the co-design activity, participants were asked to provide a summary presentation to the researchers and explain their reasoning behind the design decisions they made together.

### Data Analysis

During the PD sessions, at least 2 researchers presented and took separate notes on points discussed among groups and overall interpretations of participant reactions to ideas. After every session, the researchers collaborated to reflect on session notes and compiled a document with observations on critical insights and sentiments toward each design idea. For each PD session, a researcher also took pictures of the sketches created during the design activity and on participant handouts, which contained participant notes, feedback, and intervention rankings. We used transcripts as the primary data source, and all other collected data were used as supplementary sources. We also incorporated the co-design activity sketches as supplementary data to validate what participants discussed during the session and clarify participants’ design suggestions. The above data collection was conducted with the participant’s permission.

After organizing the collected data, we coded the first 3 PD transcripts. At least 2 researchers coded each transcript, and each code was reviewed to account for missed information. The codes were compiled into a growing codebook organized by their corresponding intervention or put into a general category. On the basis of the codebook, the remaining transcripts were then coded. If any new codes were found, they were either added to the codebook as a new code or incorporated into the definition of an existing similar code.

We then used affinity diagramming [17] to identify the emerging themes from the codes. Similar codes from all 10 interventions were first grouped together into subthemes specific to each intervention. Then, a team of researchers had a series of discussions to build larger, emerging themes across interventions that revealed the most important patient needs from the study (see Multimedia Appendix 1 [18] for the screen capture of our affinity diagramming). For example, a larger theme of patient-provider communication contained various subthemes, such as patients being unable to notify providers and improving the communication of pain. Codes that were ambiguous or needed further support were refined by referencing participant quotes from transcripts and participants’ sketches from PD sessions. After multiple group discussions, we identified 4 themes that described patients’ key needs during their ED visits.

### Ethical Considerations

We complied with the following ethical considerations. First, we recruited and obtained consent from all participants before the study sessions described in the multisite institutional review board approved by the University of Michigan (HUM00156261). Participants were informed that their participation was voluntary, the sessions would be recorded, the collected information would be deidentified and protected at a secured storage, and they would be compensated with US $100 for their participation. Second, we ensured that the collected transcript data were deidentified by replacing their names with pseudonyms, and participants’ faces in photos or videos were blurred. All collected data are stored in secured storage that requires the University of Michigan accounts. Third, all the researchers completed research compliance training such as the Collaborative Institutional Training Initiative or Program for the Education and Evaluation in Responsible Conduct of Research to become trained on best practices and ethical considerations of interacting with providers and patients.

## Results

### Overview

On the basis of the analysis of the collected data, we identified the 3 most preferred intervention ideas that participants preferred and 4 emerging themes that described the participants’ needs in the ED care process.

Across all sessions, I-4 (ie, wearable device), I-7 (ie, virtual specialist), and I-1 (ie, virtual presence of a caregiver) emerged as the most preferred intervention ideas that the participants selected to help enhance the overall quality of patient care in the existing ED process. Among 36 participants, 25 (69%) chose I-4; 22 (61%) chose I-7; and 21 (58%) chose I-1 among their top 3 preferred interventions. These preferences represent which patient needs the participants considered most important. Through I-4, participants valued provider accountability and prioritization of patient conditions in the ED waiting room. For I-7, participants valued real-time virtual interactions with providers to better use their visit time while receiving the specialized care they needed. Furthermore, for I-1, participants valued the role of the caregiver for assistance and emotional support in the ED process even if they could not be physically present.

We also report 4 emerging themes of specific needs that participants had during their ED visits. These themes encompass broader perspectives of ED patients, extending beyond the technological intervention ideas provided. While describing these needs, we explain how participants addressed or wished to address them, along with their intervention ideas. Table 4 summarizes our key findings on patient needs and participants’ suggestions for improving the intervention ideas.

**Table 4:** A summary of the 4 emerging themes based on patient needs during their ED^a^ visit, discovered across all participatory design sessions, includes a description of each patient need and participant suggestions on how to address or improve the need.

Patient needs	Description	Patients suggestions for improvement
The feeling of inclusion in the ED care process	Patients feel that their voices are not heard by providers and want to be more involved in their care process	Create more communication channels with providers (eg, Help button)
Resources to improve patient comprehension of medical information	Patients feel inadequately literate about their health and want to have reliable ways to easily understand their condition, care plan, and treatment options	Use personalized information in intervention technology to enhance patients’ understanding of their medical history
Relief of anxiety experienced by the patient over information overload and privacy concerns	Patients feel anxious because of privacy concerns and information overload in the ED. They strongly value emotional support from providers	Assist human-mediated emotional support, rather than replacing providers
Continuity in care and information	Patients perceive gaps in provider-to-provider communication that compromise care quality, emphasizing the need for improved technical infrastructure and procedures to ensure effective information delivery	Consider the accessibility of technology for different patient contexts

^a^ED: emergency department.

### The Feeling of Inclusion in the ED Care Process

Throughout the 8 sessions, it is more than evident that participants wanted to be more included in the ED process. Participants often felt unheard, dismissed, and uninformed in their interactions with providers:

[The nurses] almost like, didn’t believe me or didn’t care, until they saw like, I have to pass out in front of them before they’re going to do anything. And the second I passed out, I got moved to an actual room in the ED when I’ve been sitting in the hallway for hours.

P13

As described in the quote, participants sometimes felt that providers dismissed patient concerns unless they physically showed symptoms.

Their feeling of exclusion was caused mainly by the lack of timely and transparent communication with ED providers as they experienced providers not necessarily available to check on worsening patient conditions. Thus, it was challenging to inform providers of their health conditions:

Because there’s a lot of times if you’re on a two-hour wait and you’re in the process of having a stroke, you’re not able to get up and go back to the waiting room or the front desk, but it’d be good if something flagged that your condition was deteriorating.

P24

Because of the concern about potentially worsening conditions during the wait time, most participants favored I-4 (ie, wearable device) to track their conditions in real time and alert providers if necessary. In addition to the illustrated functionality, many participants suggested adding features such as pain-tracking and fall detection to the intervention, borrowing functionalities from other less popular intervention ideas, such as I-8 (ie, pain expression device) and I-5 (ie assistant robot). Some participants also thought that real-time tracking devices could be used to update patient priority in the posttriage stage:

If your condition is staying fine, or steady and stable and somebody else with a higher pain or priority could go first and you’re still okay, you know something like that I could see being helpful.

P3

Participants felt that by using real-time tracking data, patients in critical condition could receive medical attention as soon as needed, ultimately improving the accuracy of the diagnosis.

Despite their general support for real-time tracking of symptoms, some participants expressed concerns about how providers could use such interventions. For instance, some participants were worried that providers would ignore tracking device emergency alerts in an already hectic environment. Others also expressed concern about providers becoming overreliant on tracking devices and potentially disregarding verbal patient feedback about worsening conditions if the device’s data did not corroborate with patient concerns. Regardless of these worries, some participants pointed out that capturing real-time health data alerts could increase provider accountability in cases where patients felt ignored or misdiagnosed:

...if you’re getting this information in real-time, then...people can be held to accountability. Because you go back and you look at their chart, you look at their record, “Hey, this person was in distress, and you neglected them,” so I think in some ways, the AI could really assist in that because, again, there needs to be accountability to doctors and nurses and stuff that are just blowing you off.

P18

**Figure 4:**
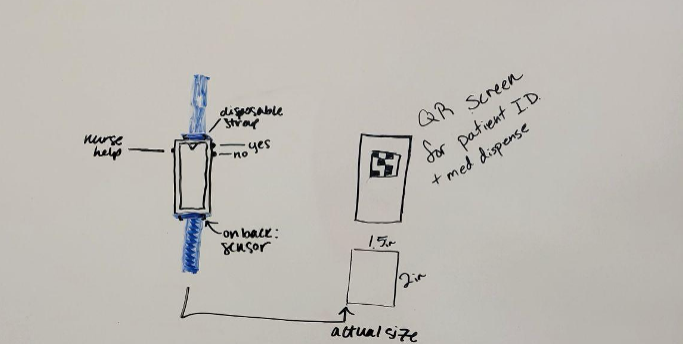
A sample drawing by a participant during a co-design activity. The drawing shows how I-4 (ie, a wearable device) could be improved with additional features such as the Nurse Help button.

Participants also advocated for additional communication channels to create a more inclusive ED care process between patients and providers. A popular idea was incorporating a “help” button that participants could use to receive immediate medical attention from a provider, which they suggested could be implemented into one or many interventions (I-2: interactive display; I-3: table-top robot; I-4: wearable device; and I-5: assistant robot). Figure 4 demonstrates a sample drawing of an improved I-4 (ie, wearable device) with a “nurse help” button on the side. Moreover, many participants indicated that the I-4 (ie, wearable device) included a way to update their condition and pain levels as they changed during their visit, and some patients mentioned that concrete pain descriptors (eg, tactile or word based) could help them better describe their pain. Participants believed that having direct communication channels, such as a “help” button, with providers could lead to better care outcomes and higher trust in their care.

As our participant pool included caregivers and patients, the role of the caregiver in patient-provider communication was also emphasized. Caregivers are especially crucial as patients advocate for children and older adults, as they can significantly help patients who cannot correctly remember or communicate their condition in the ED. Participants were receptive to the idea of incorporating I-1 (ie, virtual caregiver) into the ED process when an in-person caregiver was unavailable, and some emphasized that a caregiver list could be incorporated into patient records for cases when the patient was too incoherent to call a caregiver.

### Resources to Improve Patient Comprehension of Medical Information

Another theme we found was patients needing more information resources to improve their comprehension of care-related information. Participants expressed that their understanding of medical information required more alignment with their providers. Despite having access to their patient portal, most patients found it hard to familiarize themselves with the terminologies providers used in the ED when communicating about their condition or test results:

When I see...I think it’s [a] diagnosis or something or like conditions I have...in my chart or portal or whatever. Like it says Latin...something just saying that my knees are bad...So I wish in parentheses it could say in English...the layman’s term for that.

P25

A similar concern around patient health literacy arose when clarifying personal medical information. Participants said they often used Google (Google LLC) and other web resources to help understand their condition or diagnosis better while waiting for a provider’s consultation. One participant expressed that it could be more accessible to conduct a Google search because patients can have a baseline understanding to prepare follow-up questions for providers. However, participants also noted that when seeking medical information on the internet in this way, they could be misled into thinking their condition had severe side effects or symptoms that were not identified or considered relevant to the provider’s diagnosis.

To address the concern of potentially misleading diagnoses, participants frequently mentioned the necessity of incorporating personalized information into intervention technology to enhance patients’ understanding of their medical history. Depending on the patient’s level of health literacy, they might struggle with formulating questions or initiating conversations when communicating with their provider. In many design sessions, participants suggested that hospital-specific information can be curated based on the patient’s medical history to provide relevant and reliable search results sourced directly from the ED that might otherwise be retrieved from a Google search. Participants specifically valued the features of the I-6 (ie, ED chatbot) and I-4 (ie, wearable device) for providing personalized patient information and helping effectively facilitate conversations from the patient to provider and with the patient themselves—such technology can break down complex medical terminology so that patients can follow up with providers in a more efficient way and aid in making informed decisions about their condition:

I think that it [I-6: ED chatbot] can be helpful to the emergency room staff or doctors because they could input the information that they want you [patients] to know...so they can control the information that’s being given. Versus you [patients] googling it.

P3

Along with the difficulty in understanding medical information regarding their care, participants expressed the challenge of explaining their pain to providers. Issues with patient-provider communication arose when patients felt unable to properly convey pain levels, struggling with arbitrary scales from 1 to 10. Several participants mentioned that current pain scales do not provide adequate ways to express different types of pain:

Whenever they say on a scale of one to 10, I don’t feel pain like other people.

P15

Some participants who struggled with chronic pain also recalled that current pain scales do not take into account that people might consider different levels of pain to be “normal.” In such cases, the lack of medical information comprehension hinders patients from accurately describing their medical conditions (eg, pain) to providers. While many EDs ask about pain on a scale of 1 to 10, it is difficult to pinpoint or trace a patient’s condition over time based on this abstract measurement:

The description of the pain varies for me.... And, I do live with pain.... All the rest of the pain is still there.

P16

Some participants found the intervention idea of an I-8 (ie, pain expression device) valuable in addressing this issue, particularly for patients who cannot communicate verbally with a provider or have a language barrier. Participants particularly liked the device’s tactile capabilities as this feature could help anchor patients to a basic description or visualization of their pain to communicate with providers effectively. At the same time, some participants were also concerned about the ability of a patient to understand how a complex device works when in pain, further delaying their time in the ED and getting a proper diagnosis from providers:

If they [ED patients] are in pain already and we [the participants and researchers] have to stop and give an explanation of how to work it [a new intervention], I think it might present some difficulties, and there might actually end up being more of a barrier in terms of getting the information.

P20

### Relief of Patient Anxiety Over Information Overload and Privacy Concerns

Many of our participants mentioned patient anxiety during their ED visit as one of their top concerns. On top of being admitted to the ED, participants experienced anxiety due to various factors beyond diagnosis, including long wait times, fear of contagious diseases (especially during and after the COVID-19 pandemic), and uncertainty about the subsequent steps in the ED process. They also noted that being overwhelmed with information as a patient in the ED aggravated their anxiety. Whether from a Google search or looking at test results, patients might not be able to understand all the information presented to them. Participants thought it was essential to consider the types of information that would be accessible—such as vitals or pain levels—to patients, providers, or both:

Do [patients] know how to read it? Do they know how to interpret the information that’s given to them? Too much information for a patient can be bad. Then their mind wanders because they don’t know.

P30

Potential anxiety induced by overwhelming information brought different perspectives regarding how much information should be displayed in the intervention ideas. Participants wanted to be informed but felt too much information could be unnecessary for patients who did not understand medical terminology:

I think that’s a hard one. Because some people—it doesn’t stress them out to know, and they like to know, other people can like to say their heart rates [are] high, but it can make it worse because then they’re anxious about it.

P13

Participants across sessions discussed different scopes of information access available for each intervention idea. For example, patients might be presented with a fundamental view that only shows whether their vitals were in a normal range. At the same time, providers might have more detailed access to numeric values and other data that could help track patient conditions over time. Participants suggested this could be paired with alerts on the I-4 (ie, wearable device) for the patient and provider, or providers only, to notify hospital staff in an emergency and better support care priority in the waiting room. Some participants even considered extending this idea for the entire duration of a patient’s ED visit. Overall, participants suggested informing providers of a patient’s condition would help mitigate patient anxiety in an ED setting.

The other factor that contributed to patient anxiety was related to patient privacy. Some participants felt that their health information was exposed to others in the waiting room when providers periodically checked in with them. Participants did not feel their privacy was protected during these discussions and thus felt their rights were being violated. Participants also attributed this feeling to more functional ED issues such as the waiting room’s size, layout, structure, and overall ED design. With a small waiting room and open, shared space for beds in the ED, participants felt uncomfortable about exchanging confidential health information between patients and providers in open environments and public areas:

I heard everything that was going on. I could have told you what was going on....Because of our health and our health being we have rights for nobody to know.

P16

P20 also had a similar experience:

I’m hearing people’s whole names being called out in this huge waiting room. I felt like the confidentiality was being breached because I knew that person was there getting care.

To address this issue, many participants advocated for existing ED processes to be remediated at the structural level. This included the addition of more ED beds with closed walls or partitions, private spaces in the waiting room, and priority-based interventions. Overcrowded EDs induce long wait times and quickly fill up space in the waiting room where patient information is highly vulnerable to others. Some participants also suggested interventions, such as I-2 (ie, interactive display) and I-5 (ie, assistant robot), should avoid displaying personal information to protect patient privacy, primarily because these interventions would be concentrated in the waiting room.

To alleviate anxiety induced in general by multiple factors during ED visits, many participants stressed the importance of having human providers as an aspect of emotional support for patients in the ED care process. When presented with ideas that limited patient-to-provider interactions (eg, I-3: table-top robot and I-10: AI caller), participants expressed how technological interventions should support human providers rather than replace them. For this reason, interventions involving AI or robot technology were not popular with participants because they could have limitations in understanding patient requests or evaluating a patient’s condition because of the lack of sophisticated technology in this space. Participants further highlighted empathy as another critical factor to consider because technology in the form of a robot would not convey as much sympathy and support as a human would in a stressful ED situation. Many participants thus preferred to have human support for alleviating their anxiety along with technological interventions because humans can communicate medical information and reassure patients in a manner that helps in coping with an anxiety-inducing environment:

I don’t think people would have a lot of confidence in [I-5: assistant robot] either. You know what I’m saying—it needs to be people who still like to interact.

P18

### Continuity in Care and Information

During group discussions, many participants shared their experiences with discontinuity in care and information. On the basis of their experience, participants perceived that discontinuity might have occurred due to the handoff process within the care team. They noted that the involvement of multiple providers in their care team meant that information would often get lost during handoffs. Because of such missing information, some participants experienced discrepancies in provider-to-provider communication that caused patient confusion:

Even a stay in the hospital, the doctors just rotate and roll over.... The first doctor said they wouldn’t consider [discharging] until it [the protein level] goes below 10,000. Then the next doctor said, “Oh no, I’m not going to even think about it unless it’s below 500.” So, each time the shift changed—they told us something different.

P10

Such discrepancies in care also occurred between hospitals. For instance, P27 shared her experience when she received a different diagnosis in the second hospital:

So if he [P27’s son] would have asked for a second opinion, at that point, you’re leaving me in a lot of pain, and I still don’t know what’s going on. If he could have asked for that second opinion at that point, maybe he would have been diagnosed with a real issue five days earlier than he was.

P27

Reflecting on those situations where information discontinuity had occurred, most participants favored the I-7 (ie, virtual specialist) to improve the breakdowns. They insisted that for interventions such as I-7 (ie, virtual specialist), an ED physician should be present when the patient talks to the specialist so that the ED care team could have updated information. They envisioned that bringing specialists directly to the ED care process would improve diagnosis and documentation in patient care records with the second opinion. Participants also expressed the need to allow for secure but easy information sharing with providers outside the hospital when external providers were invited to the ED care process. For instance, a global access health care platform could be used across hospitals for patient medical data transfer. Therefore, they thought that I-7 (ie, virtual specialist) could be expanded to include medical professionals beyond certain specialists:

I think it depends on the context, the question, but for certain questions, it could be any nurse, it could be a PA [physician assistant], or someone who had familiarity with the patient. Based on the level of question, it may need to be a provider. Or a pharmacist.

P27

To promote continuity of care, participants commented that the technological interventions should be designed in an accessible way for different patient situations. For example, some patients wanted to use their existing wearable devices like smartwatches or fitness trackers instead of wearables provided by the ED to integrate live vitals tracking with historical data accurately. They also suggested that the I-6 (ie, ED chatbot) could be offered as an app for mobile devices that patients and caregivers could download when they arrived at the ED and use throughout their ED stay and after their ED care. These participants expected that using interventions with patients’ devices would ensure that patient-collected health data would be used to its fullest extent during care in the ED and would allow providers to glimpse the patient’s complete health profile over time rather than only view current condition. On the other hand, some other participants emphasized the need for care continuity for those who may not have the same access to technology (eg, smartphones and smartwatches) or may have different amounts of previously collected health data, for instance, patients with chronic illnesses may have more personal health data tracked than those without. Thus, it is important that intervention use did not become a privilege reserved only for a subset of patients who had access to certain technological and data resources:

I think that one, look at the person that comes in without any communications, no phone or no computer, you know, nothing.... I often go into situations and emergencies without any kind of feature.... I carry a phone—an old-fashioned flip phone with me. I can’t access some of those things.

P20

Participants’ desire for continuity of care was also shown during their co-design activity when they worked to improve the intervention designs. They envisioned an integrative, connected system where multiple intervention ideas were integrated with the current health portal or text notification system to flow health-related information continuously. For example, they envisioned a tablet that could be used for I-7 (ie, virtual specialist), displaying vitals synchronized with I-4 (ie, wearable device) or the patient’s health device (Figure 4). Through such integration of features, participants envisioned inventions to enhance the continuity of care and information and to address their complex challenges in the ED care process. Although a few participants felt concerned that such an integrated intervention could delay ED stay time or contribute to patient information overload, many still thought our suggested interventions could be integrated with the existing patient portal and offered in an app format on various devices.

## Discussion

### Design Implications for Patient-Centered Interventions in ED

On the basis of the PD sessions, we identified ED patients’ needs in the ED care process and expectations for future technological interventions. This section discusses how these findings may lead to design guidelines for more patient-centered interventions in EDs.

First, technology-based *interventions should address patients’ dynamic needs to promote continuity in care*. Our participants found that their needs could be met by more than one intervention idea or multiple specific features because their complex needs may change depending on where they stand in the ED care process. For instance, many participants suggested integrating critical functions into one system in the co-design activity so that the system could provide necessary and relevant information to them, from arrival at the ED to postvisit follow-up. This desire was evident when some participants envisioned an integrated system of 2 different intervention ideas (I-7: virtual specialist and I-4: wearable device) that could track patients’ status and share data with specialists during the diagnosis process. Such integrated interventions may also be used to support issues in patient-provider communication. Our findings demonstrated that patients lacked sufficient information and interactions with ED providers. Thus, our participants shared how integrated interventions may help fill this information gap by providing relevant communication channels or resources based on patients’ dynamic needs during ED visits. However, mere integration of systems may lead to additional challenges for patients. As some of our participants highlighted, numerous resources may cause information overload that can lead to patient anxiety. In another case, inviting outside providers (ie, specialists) would require extra documentation, potentially resulting in additional patient burdens. To incorporate a patient-centered integrated system in the ED, we suggest that the system be capable of adapting to patients’ needs throughout the ED care timeline rather than simply adding features into one system. Existing technologies are primarily designed to provide specific information to patients in a certain part of the ED process (eg, a mobile app for an interactive report [7] to inform patients about their real-time progress and care plans during their visits). Such technologies can be integrated with other features, such as I-10 (ie, AI caller) for after-visit support, to address the dynamically evolving needs of patients that extend beyond their stay in the ED. An integrated system that supports patients starting from triage to beyond their discharge would provide timely, relevant information to patients based on their status within the ED care process. This integration approach to promoting continuity in care resonates with prior studies that designed a multicomponent app for patients and caregivers, the Bone Marrow Transplant Roadmap [19]. The app included components that reflect the information needs of patients and caregivers during their clinic visits, such as an overview of the criteria needed for discharge, real-time laboratory results, personalized medication lists, and educational materials. This app was further expanded to include outpatient settings to better support caregivers [20]. Similar to these multicomponent approaches, we suggest the integration of various features to better meet ED patients’ dynamic needs during or even after their ED visits.

Second, *interventions should account for both the amount and timing of information provided to patients.* Our participants shared their challenges in accessing the appropriate amount of information during their ED visits. They felt excluded from the care process when they received insufficient information, while overwhelming information heightened their anxiety. Thus, participants suggested that the patients should have the decision to choose the timing and extent of information they receive for applicable interventions, such as opting for partial, complete, or no information access. One example of this suggestion was using a chatbot to provide patients with personalized care information. On the basis of the participants’ ideas, I-6 (ie, ED chatbot) could be further developed to offer relevant information depending on the steps patients undergo in the care process and their current health condition. Prior studies have shown that chatbots can provide more accessible and relevant information to patients [21]. In cancer contexts, a chatbot provided answers to patients with breast cancer with similar satisfaction as physicians [22], while another chatbot collected patient-reported symptoms during chemotherapy and reduced ED visits and unscheduled hospitalizations [23]. While these studies presented the opportunities for chatbots to provide information to patients, our findings suggest more careful considerations on the timing of providing information to patients. The need for timely and appropriate information resonates with the findings of a prior study [24], which describes how temporal dependency can delay the information that ED patients need. As ED medical activities often depend on previous activities, the information patients need or want is usually delayed. Without having relevant information during the ED process, patients may experience communication challenges with ED providers, such as different perceptions of what information is critical to ED care [25]. Therefore, as our participants suggested, interventions should provide ED patients with optimized information relevant to the current step of the care process or options to control the amount and timing of information they expect to receive.

Third, *interventions should empower patients to be more active in their care process for better patient safety and care quality*. Our findings highlighted participants’ need for patient autonomy in ED care. Unlike other patient care settings, the ED context is particularly intimidating for patients because ED visits are unexpected and urgent. Each visit may be different from the previous one. However, we found that participants in our study were eager to take a more active role in the ED care process. In the PD sessions, the need for empowerment was evident when most participants suggested features that allowed them to communicate assertively with providers about their symptoms. For instance, although I-4 (ie, wearable device) was initially designed to track patients’ condition so they would not feel forgotten while waiting, many participants wanted to have another function, similar to a “help” button, to request support more actively from providers when their conditions worsened or changed. This suggestion indicates how ED patients want to be more active stakeholders in the ED care process rather than be considered passive stakeholders who are just being assessed for their symptoms. Patient-empowering interventions are implemented on the basis of the prior studies’ insights from existing interventions, such as an inpatient portal [26] and a smartphone app [27]. For instance, a smartphone app called MySurgery [27] was developed to provide information about surgery risks and practical step-by-step advice for each risk. The app demonstrated a significant ability to enable patients to engage actively in discussions regarding their care and adopt behaviors related to their safety. Similarly, ED patients can be empowered with relevant information and advice, such as explaining medical terms or notices about the next step in the ED care process. This need for patient empowerment resonates with previous studies on shared decision-making in the ED [28,29]. These studies identified that ED patients want some degree of involvement in medical decision-making. Extending this line of study, we found contextual nuances and more concrete examples of ED interventions to promote patient involvement. For instance, a review study showed the opportunity for wearable devices to monitor patient vital signs and provide the collected data to providers to improve decision-making [30]. Our findings suggest how ED patients perceive such wearable devices as an opportunity to be more actively involved in the care process rather than letting the devices passively collect data. Therefore, we suggest that future ED interventions be designed to empower patients to be included in shared decision-making.

Fourth, *interventions should optimize human resources, depending on patients’ needs*. We identified that some issues cannot be addressed through technological interventions alone. These issues may be related to distress or emotional needs of ED patients. For instance, our participants preferred human support rather than interactions with technical tools in alleviating their feeling of anxiety during ED visits. This preference implies that ED patients can manage their stress through human interactions, although the suggested interventions, such as I-5 (ie, assistant robot), can still provide medical information. Moreover, human resources for ED patients can include those who are not ED providers. Many participants expressed their need to connect with informal caregivers (eg, family members), specialists, or other providers during their ED visit. Their needs were particularly evident when patients visited the ED once because they did not know about the ED care process. On the basis of their previous visits, patients learned that some external resources (eg, family members and specialists) could provide more information to help assess their conditions or, in the case of family members, provide emotional support during the ED visit. This finding indicates that ED patients leverage resources they are more familiar with in the intimidating and unfamiliar ED settings. Prior studies in the medical literature have shown the opportunities and challenges of telehealth for patients [31-33]. However, those identified challenges (eg, unsteady or poorly framed video [33]) and strategies to overcome them may not be implemented in the fast-paced ED care process. Depending on the patient’s current conditions, the right timing (eg, when a specialist can be contacted) and method (eg, simple video call or additional camera to show the patient’s status) for providing external resources to patients should be determined. Thus, designing patient-centered interventions should optimize human resources to address ED-specific challenges, as we identified in our study. It will be critical to identify when and how interventions should provide external resources to patients. For instance, some of our participants wanted to be connected with specialists when they met ED providers after examinations so that they could have second opinions on their symptoms.

Finally, *interventions should be designed considering patients’ perspectives on implementation*. Through co-design activities, we identified how our participants expected intervention ideas to be implemented to address their needs better. However, their expectations could differ from actual implementations, potentially conflicting with what providers or the hospital expect. In such cases, it is critical to mitigate those expectations to implement patient-centered interventions. For instance, one of the potential reasons for our participants’ preference for I-7 (ie, virtual specialist) was the assumption that specialists are available to have on-demand consultations. In contrast, in actual implementations, it would require other resources (eg, cost, time, and human resources) to implement a system to become connected with specialists. As a prior study shows, ED providers must modify their work practices to adopt the new interventions [34]. Moreover, some of our participants pointed out the accessibility of devices that ED patients may not have. While mobile device ownership is high among ED patients [35], our suggested intervention ideas involved devices that patients may not have, such as wearable trackers. Thus, it is essential to consider how ED patients perceive the implementation of technological interventions in the ED.

### Limitations

This study has some limitations. Given their qualitative nature, the findings are specific to the context we examined. Providers and patients from other EDs may face different challenges. However, our findings will contribute to identifying potential interventions that address such challenges through a PD approach. In addition, we recruited participants with varying backgrounds from 2 ED units (pediatric and adult EDs), which could help with the study’s generalizability. Another limitation is that we did not distinguish participants’ previous experiences as ED patients or caregivers. While the ED experiences of patients and caregivers do not have noticeable differences, future work can explore caregiver-specific perspectives on interventions designed to address their challenges in the ED care process. Finally, we only presented concepts, not interactive system prototypes. Therefore, participants may not be quite familiar with some intervention ideas. They could only imagine how an intervention might work but could not see the real system or product. However, storyboards are a common Human-Computer Interaction methodology for eliciting user needs in the early system design stage. They are useful for validating user needs and expectations before implementing a system in a complex environment similar to an ED.

### Conclusions

In this PD study, we invited ED patients to design sessions to assess their needs in the ED care process and co-design technical interventions. On the basis of the analysis of collected data, we identified 4 themes related to ED patients’ needs: the feeling of exclusion from the ED care process, limited resources for patient comprehension of medical information, anxiety about overwhelming information and privacy, and discontinuity in care and information. Concerns were also expressed about the accuracy of the diagnosis. These findings also informed us to develop guidance for designing future technology-based patient-centered interventions to improve the diagnostic process in the ED. To refine the identified design guidelines, we aim to conduct a PD study with ED health care providers. By identifying similarities and differences between ED patients and providers, we expect to present concrete design guidelines for technological interventions to support the ED diagnostic process and patient safety.

## Appendix

Multimedia Appendix 1 A screen capture of our affinity diagramming. We used the Miro board [18] for affinity diagramming.

## Abbreviations

AI: artificial intelligence
ED: emergency department
PD: participatory design

## Data Availability

The datasets generated and analyzed during this study are not publicly available because the consent form for the participants stated that the data could only be accessible to the research team.
