# Supplementary material for: Designing Patient-Centered Interventions for Emergency Care: Participatory Design Study
Source: JMIR Form Res. 2025 Feb 12;9:e63610. doi: 10.2196/63610 (PMC11888112; doi:10.2196/63610)
Supplement: Multimedia Appendix 1 [file formative_v9i1e63610_app1.pdf]

Patient-provider communication

19. Provider needs to have more empathy and support

1: Patient unable to communicate with provider

3: Impact of caregiver absence

4: Ways for patient to notify providers

3: Notifying providers in emergency situations

4.1.a. A. Informing providers

6. Communication Barriers between patients and provdiers

9. Patient-provider trust and communication

8. Patient-provider communication

8. Improve Communication of Pain - patient engagement

8. Improve Communication of Pain - simulations, tactile description, comparison with past

Insufficient ED capacity/providers

7. Lack of ED staff

5: Robot can assist providers with tasks

Patient Anxiety

5. Patient feels anxiety

2: Display of personal information

3: Can provide distraction/support in the waiting room

6. Anxiety

Changes to current ED/Process

7: Creating Physical Privacy

1: Confusion about general ED proccesses

2: Communication of general ED proceses

3: Availability of charging ports

Care quality

16. Patients want continuity of care

7: Continuity of Care

9. Continuity of Care

10. Continuity of Care - AI caller

7: Care Quality

4: Care Quality

8. Patient Priority

4.3. Real Time Condition Tracking

Concerns of disease transfer

10. Disease transfer

2: Patients worry about disease transfer

Integrating technologies

4: Integrating with Existing

1: Potential for integration on hospital device

3: Alternative device/function to the robot

6. Technological Integration

9. Integration of Technology

5: Alternative device/function to the robot

Tech inclusivity & customizability

6. Patient has a language barrier

7. Display can have translation features

6. Chatbots should be inclusive and customized

9. Information Accessibility/ inclusive and customized

10. Personalized and Opt-in Services

1. Optional use

3: Patients have option to use robot

Long waiting

9. Wait times and ED stay length can be lengthy

4. Worsening patient condition

2. Giving nurses more medical authority

20. Provide essential items for comfort and care

2: Entertainment in the ED

3: Can provide distraction/support in the waiting room

4: Reducing Wait Time

7: Reducing Wait Time

9. concern about: may increase wait time

Control over the intervention

3: Negative drawbacks of robot implementation

4: Who should see vitals/be notified

4: Who should get access

5: Limitations of robot implementation

7: Who should get access

1. Supply-demand

Patient privacy needs

1. Patient needs privacy

1: Patient privacy between caregiver

2: Display of personal information

2: Privacy by divider separation

5: Limitations of robot implementation

7: Privacy Considerations

Intervention is ineffective/feasibility

1. Intervention increases provider workload

4: Accuracy Concerns

5: Limitations of robot implementation

8. Feasibility

8. do not trust new tech, not useful

9. Feasibility

10. feasibility

10. do not trust new tech, not useful

5: Preference of human communication

6. tech preference - prefer human

10. Perfer human (in Automated Communication)
